# Thermodynamics of Morphogenesis: Beading and Branching Pattern Formation in Diffusion-Driven Salt Finger Plumes

**DOI:** 10.3390/e27020106

**Published:** 2025-01-22

**Authors:** Hisashi Ozawa, Sayaka Murayama-Ogino, Axel Kleidon

**Affiliations:** 1 Graduate School of Advanced Science and Engineering, Hiroshima University, Hiroshima 739-8521, Japan; 2 School of Integrated Arts and Sciences, Hiroshima University, Hiroshima 739-8521, Japan; 3 Max Planck Institute for Biogeochemistry, 07701 Jena, Germany; axel.kleidon@bgc-jena.mpg.de

**Keywords:** thermodynamics, pattern formation, energy accumulation, energy dissipation, non-equilibrium systems, morphogenesis

## Abstract

Spontaneous pattern formation is a universal phenomenon that occurs in purely physical systems, biology, and human societies. Salt fingering due to differential diffusion of heat and salt in seawater is a typical example, although the general principle that governs pattern formation remains unknown. We show through simple experiments injecting a salt solution into a sucrose solution of equal density that a salt finger exhibits characteristic pattern transitions depending on the injection flow rate. When the rate increases, a linear finger starts meandering, branching, and multiple branching, whereas when the rate is decreased, it produces a beading pattern. These morphological instabilities and associated pattern formation are caused by a local accumulation of kinetic energy that minimizes the flow resistance and maximizes the energy dissipation in the final steady state. We suggest that this energy accumulation mechanism governs a wide variety of pattern formation phenomena in non-equilibrium systems, including morphogenesis of abiotic protocells.

## 1. Introduction

Spontaneous formation of regular and ordered patterns from initially homogeneous distributions of materials in systems far from thermodynamic equilibrium has long attracted interest of researchers in various fields [1,2,3]. Typical examples include the formation of cellular patterns in thermal convection [4], crack pattern formation in stressed solids [5], dendritic growth of crystals [6,7], vortex formation in front of granular flows [8], and morphogenesis during the growth of living organisms [9]. These phenomena seem to possess common characteristics, i.e., the spontaneous formation of ordered and coherent patterns in systems far from equilibrium conditions and the resultant rapid and efficient recovery to equilibrium. It would, therefore, seem to be a unified law that governs these pattern formation phenomena in general. However, no complete theory that can explain the mechanism of pattern formation has yet been presented [1,2,3,10,11,12]. Here, we propose that all of these patterns are formed by the local accumulation of kinetic energy, which minimizes the flow resistance and maximizes the dissipation of total available energy, and we confirm this hypothesis with a series of laboratory experiments on finger-type convection patterns.

**Figure 1:**
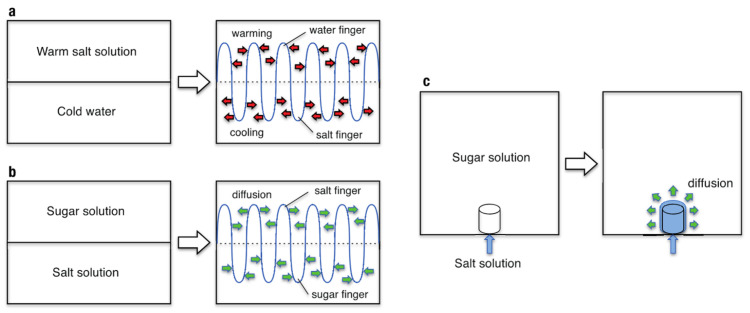
Schematic illustrations of double-diffusive convection. (**a**) Warm salt solution on cold fresh water, resulting in ascending water fingers and descending salt fingers. (**b**) Sugar solution on salt solution, resulting in ascending salt fingers and descending sugar fingers. (**c**) Salt solution injection into sugar solution, leading to an ascending salt finger (this study). The densities of the two solutions are initially set to be equal.

Salt fingering, finger-type convection caused by the difference in diffusivities of heat and salt in seawater, is a typical pattern formation phenomenon that occurs in the global ocean [13,14]. Figure 1a shows a setting where a warm salt solution lies on cold fresh water of nearly equal density, mimicking a situation of subtropical seawater. Since the diffusivity of heat, *D*_h_, is much higher than the diffusivity of salt, *D*_s_ (*D*_h_ ≈ 100 *D*_s_), the salt solution above the interface cools and becomes denser via preferential heat diffusion, whereas the fresh water below the interface warms and becomes lighter over time. This unstable density distribution thus leads to descending “salt fingers” and ascending “water fingers,” emerging from the interface in mutually opposite directions, as shown in Figure 1a [14]. A similar type of convection occurs when a sucrose (sugar) solution lies on a salt solution of the same density [15,16,17,18]. Since the salt diffusivity is approximately three times higher than the sucrose diffusivity, preferential salt diffusion makes the overlying sucrose solution denser and the underlying salt solution lighter, leading to descending “sugar fingers” and ascending “salt fingers” from the interface (Figure 1b). In both cases, the finger motion is driven by buoyancy/gravity force caused by preferential diffusion of heat or salt. If the finger were too thick, diffusion would be inefficient to drive the finger motion, whereas if it were too thin, viscosity would suppress the motion. We therefore expect that each finger may adjust its size and shape so as not to significantly increase the viscous force, while maintaining the diffusion efficiency for the finger motion. Although several experimental studies have been conducted on salt fingers, little is known about the shape and stability of a single finger because numerous fingers emerged simultaneously from the interface in previous experiments [14,15,16,17,18,19], preventing a detailed analysis of the behavior and motion of a single finger.

**Figure 2:**
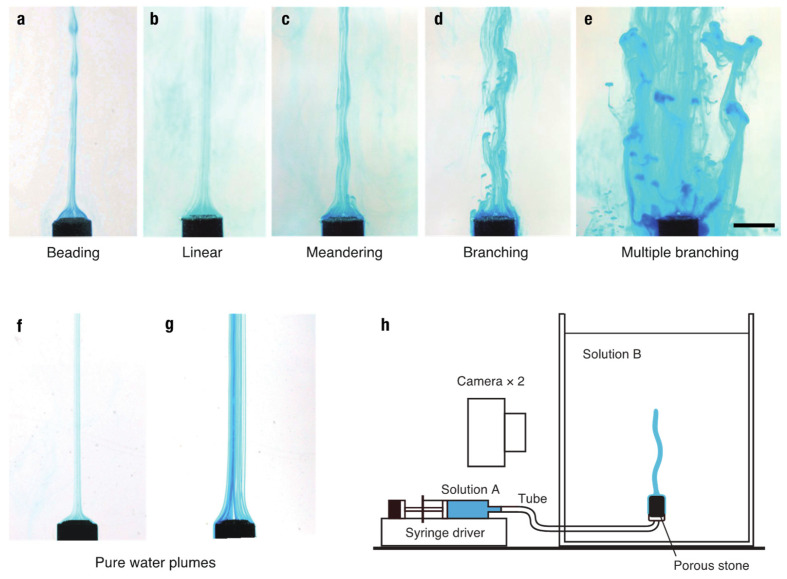
Five characteristic types of salt fingers. (**a**) Beading, (**b**) linear, (**c**) meandering, (**d**) branching, and (**e**) multiple branching patterns. The injection flow rates, *Q*_in_, are 0.83, 1.7, 8.3, 17, and 83 mm^3^ s^−1^, from the left (**a**) to the right (**e**). The initial solution densities, *ρ*_init_, are 1050 (**a**) and 1100 kg m^−3^ (**b**–**e**). Scale bar, 10 mm. (**f**,**g**) Injection of pure water into sugar solution (*ρ*_init_ = 1100 kg m^−3^) results in linear water plumes over a wide range of *Q*_in_ between 0.83 and 83 mm^3^ s^−1^. (**h**) Schematic of the experimental apparatus. Dyed solution A (salt solution) is injected into solution B (sugar solution) of equal density through a porous stone at a constant rate, *Q*_in_, using a syringe driver.

To investigate the growth process and stability of a single finger, we conducted idealized experiments in which dyed salt solution was injected into sucrose solution of the same density from an injection point at a constant flow rate (Figure 1c). The injected salt solution was subject to buoyancy force due to preferential salt diffusion and ascended from the injection point in the form of a finger-like plume. We investigated the growth process and patterns of the produced finger by varying the injection flow rate, *Q*_in_ = 0.17–100 mm^3^ s^−1^, and the initial density of the two solutions, *ρ*_init_ = 1010–1200 kg m^−3^. The injection rate was controlled with a syringe driver, and a porous stone was used to reduce the initial flow velocity and diminish initial perturbations. The porous stone was saturated with salt solution to avoid mixing with the sugar solution inside the porous stone. The growth process was monitored with two digital video cameras, and the mean velocity and diameter of a finger in its steady state were measured through analysis of the digital video images (see Appendix A for further details). A schematic of the experimental apparatus is shown in Figure 2h.

## 2. Results

**Figure 3:**
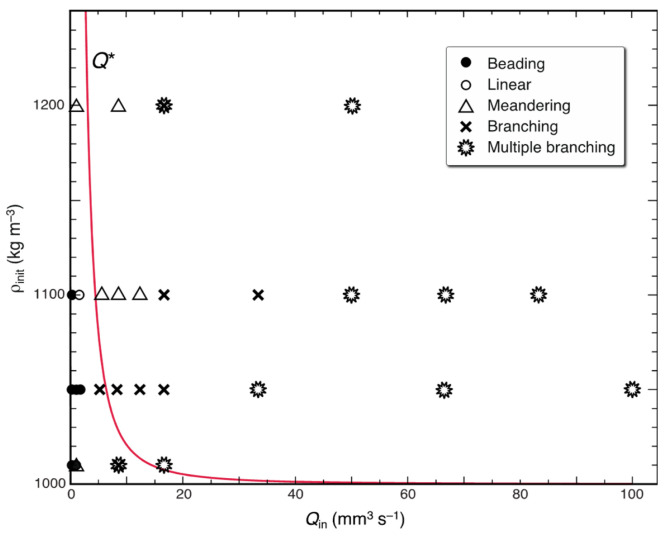
Dependence of the finger type on the injection flow rate, *Q*_in_, and the initial density of the solutions, *ρ*_init_. Symbols: ●, beading; ○, linear; △, meandering; ×, branching; 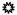, multiple branching. The solid (red) line indicates the flow rate for the maximum-velocity finger, Q*, as a function of the initial density, *ρ*_init_.

We observed five characteristic salt finger patterns in our experiments: (a) beading, (b) linear, (c) meandering, (d) branching, and (e) multiple branching patterns, as shown in Figure 2. Each pattern depends on the injection flow rate and the initial density of the two solutions (Figure 3). Under the same density conditions, a linear finger starts meandering and forming branches and multiple branches when the injection flow rate is increased, whereas it produces a beading pattern when the flow rate is decreased. The beading pattern consists of a train of small droplets connected through a thin plume, all of which ascend in a “bead-on-string” form (Figure 2a, Video S1). The beads are produced when the diameter of a plume becomes thinner than a certain size at a few centimeters above the injection point. They eventually split into discrete droplets in their final states (Video S1). This transition is referred to as *beading instability*. By contrast, branching occurs at marginal parts of a meandering finger via “budding” (Figure 2d, Video S4). We call this transition *branching instability*. As the flow rate increases further, the finger repeats branching and tends to be an assemblage of branched fingers, i.e., “multiple branching” (Figure 2e, Video S5). It should be noted that when we inject pure water into sucrose solution, no such instability occurs over a wide range of flow rates (0.83–83 mm^3^ s^−1^) despite the stronger buoyancy acting on the water finger (Figure 2f,g). This clearly shows that both instabilities are of a diffusion-driven type and do not occur in conventional-type convection where diffusion always weakens fluid motion rather than enhancing it. The Reynolds number of the finger motion is in the range of Re = *d v*/*ν* = 5–100, where *d* is the diameter, *v* is the ascending velocity, and *ν* is the kinematic viscosity. Thus, the finger motion is essentially turbulent and cannot be considered as a simple laminar flow.

Beading instability leads to a reduction in the surface area of a thin finger, and this resembles the Plateau–Rayleigh instability known to occur in a thin fluid column under the effect of surface tension [20,21]. However, there is no surface tension in our case, but rather a viscous shear layer between the ascending finger and the surrounding fluid. This instability is therefore a dynamic one caused by velocity shear rather than a static one due to surface tension. Both instabilities nonetheless lead to a reduction in the surface area of a thin finger. By contrast, branching instability leads to an enlargement of the surface area. With this process, the number of fingers increases, and the specific surface area of the fingers increases drastically. We find that branching instability occurs when the injection flow rate is increased, whereas beading instability occurs when the rate is decreased.

**Figure 4:**
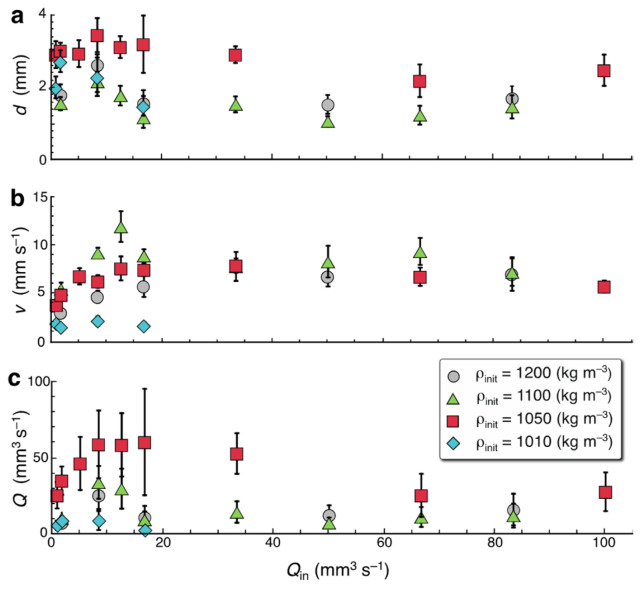
(**a**) Diameter, (**b**) ascending velocity, and (**c**) ascending flow rate observed for salt fingers as function of the injection flow rate. Measurements are made with digital image analysis, and the error bars indicate standard deviations.

Figure 4a–c show the diameter, *d*, ascending velocity, *v*, and ascending flow rate, *Q* = *π* (*d*/2)^2^ *v*, respectively, observed for salt fingers as a function of the injection flow rate, *Q*_in_. For each initial density, as the injection flow rate increases, the diameter increases, reaching a maximum before branching, and then decreases slightly after branching. A similar trend is observed in the ascending velocity: *v* increases with *Q*_in_ until branching starts and then decreases gradually after branching. The ascending flow rate of a single finger also shows a similar trend: *Q* increases with *Q*_in_ until branching and then decreases after branching. The somewhat large error bars in the measured diameters and velocities are due to turbulent fluctuations in the ascending fingers (Figure 2).

## 3. Discussion

### 3.1. Theoretical Finger Model

On the basis of our experimental results, we considered how a salt finger attains a stable shape using a simple theoretical model. We assumed a columnar shape for a finger and a steady state in which the force balance between buoyancy and viscous force and the salt balance between advection and diffusion of salt molecules are maintained. For simplicity, we neglected the effect of the slow diffusion of sucrose molecules in this attempt.

The salt diffusion flux, *J*_d_, from a finger with salt concentration *C*_f_ to the surroundings per unit length per unit time is given as follows:

(1) $$J_{d}\;=\;-\pi d\cdot D_{s}{\left.\frac{\partial C}{\partial r}\right|}_{r=d/2}=\;\pi d\cdot D_{s}\frac{C_{f}}{\delta_{d}},$$

where *d* is the diameter, *D*_s_ is the diffusivity of salt molecules, *C* is the volumetric salt concentration (salinity), *r* is the radial distance from the center of the finger, and *δ*_d_ is the thickness of the diffusion boundary layer, defined as $\delta_{d}\;\equiv \;-C_{f}/{\left.(\partial C/\partial r\right|}_{r=d/2})$. Conversely, salt is transported into the finger by advection due to the ascending motion. The salt advection flux into the finger, *J*_ad_, per unit length per unit time is given by the following equation:

(2) $$J_{\mathrm{ad}}\;=\;\pi {\left(\frac{d}{2}\right)}^{2}\beta v\;=\;\frac{\pi }{4}\beta \;d^{2}v,$$

where *v* is the ascending velocity, *β* = *C*_0_/*L* is the mean gradient of salinity, *C*_0_ is the initial salinity, and *L* is the characteristic length of the finger (system size). In a steady state, the diffusion flux should be balanced by the advection rate (*J*_d_ = *J*_ad_), and we obtain the following equation:

(3) $$C_{f}=\frac{\beta \delta_{d}d}{4D_{s}}v.$$

Let us next consider the force balance on the finger. Because of salt diffusion, the salinity in the finger, *C*_f_, is lower than the initial salinity of the solution, *C*_0_, injected through the porous stone. The density of the surrounding sucrose solution, however, remains nearly the same as that of the initial solution. Thus, the density of the solution in the finger becomes less than that of the surrounding fluid by the amount Δ*ρ* = *C*_0_ − *C*_f_, and the finger is subjected to the net buoyancy force (buoyancy minus gravity force) from the surrounding fluid. The net buoyancy force, *F*_b_, exerted on the finger per unit length is given as follows:

(4) $$F_{b}=\pi {\left(\frac{d}{2}\right)}^{2}\cdot \Delta \rho \cdot g=\frac{\pi }{4}\;\left(C_{0}-C_{f}\right)g\;d^{2},$$

where *g* is the gravitational acceleration. Conversely, a finger moving upward with the velocity (*v*) is subjected to the viscous drag force in the downward direction. The viscous drag force, *F*_v_, per unit length is given by the following equation:

(5) $$F_{v}=-\pi d\cdot {\left.\mu \;\frac{\partial v}{\partial r}\right|}_{r=d/2}=\;\pi d\mu \frac{v}{\delta_{v}},$$

where *μ* is the viscosity and *δ*_v_ is the thickness of the viscous boundary layer, defined as $\delta_{v}\;\equiv \;-v/{\left.(\partial v/\partial r\right|}_{r=d/2})$. In a steady state, the force balance is held (*F*_b_ = *F*_v_), and we obtain the following equation:

(6) $$(C_{0}-C_{f})\;g\;=\;4\mu \frac{v}{\delta_{v}d}.$$

**Figure 5:**
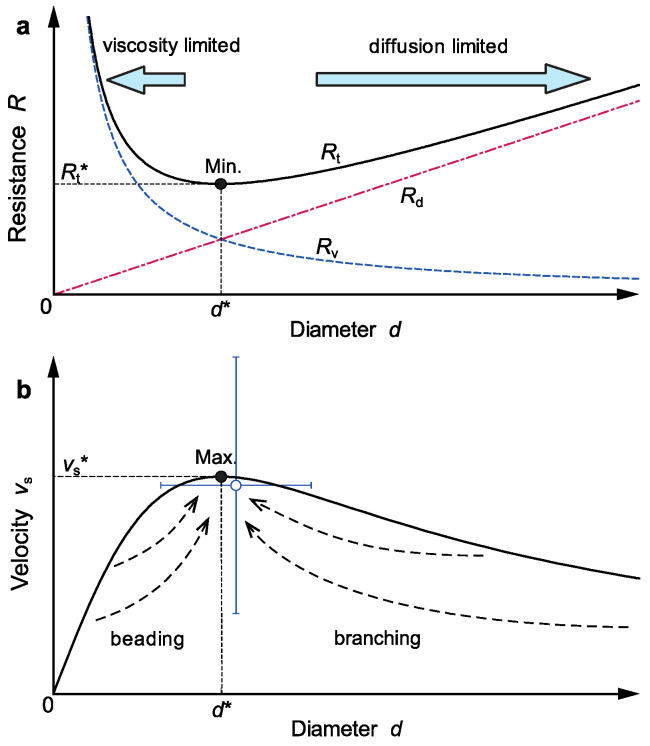
(**a**) Flow resistance due to viscosity, *R*_v_, that due to salt diffusion, *R*_d_, and the total resistance, *R*_t_ = *R*_v_ + *R*_d_, as a function of the diameter, *d*. (**b**) The steady ascending velocity, *v*_s_, as a function of the diameter, *d* [Equation (7)]. The open circle (○) indicates the mean velocity and mean diameter of the linear and meandering fingers observed in the experiments, and the error bars indicate the standard deviations. The dashed arrows show schematic transitions of salt finger patterns.

Substituting Equation (3) into Equation (6) and eliminating *C*_f_, we obtain the ascending velocity of a salt finger in the steady state, *v*_s_, as a function of the diameter, as follows:

(7) $$v_{s}\;=\;\frac{C_{0}\;g}{\frac{4\mu }{\delta_{v}d}+\frac{g\beta \;\delta_{d}d}{4D_{s}}}=\;\frac{C_{0}\;g}{R_{v}+\;R_{d}}$$

where *R*_v_ = 4*μ*/(*δ*_v_*d*) represents the resistance to the finger’s ascending motion due to viscosity and *R*_d_ = *gβδ*_d_*d/*(4*D*_s_) represents that due to salt diffusion. We can see from Equation (7) that the numerator on the right-hand side, *C*_0_ *g*, represents the overall driving force for the finger’s ascending motion, and the denominator, *R*_v_ + *R*_d_, represents the sum of the resistances due to the viscosity and diffusion processes. The viscous resistance is inversely proportional to the diameter (i.e., decreases with *d*), whereas the diffusive resistance is proportional to the diameter (i.e., increases with *d*). The viscous resistance limits the ascending velocity of a very thin finger, while the diffusive resistance limits the velocity of a very thick finger. The total resistance, *R*_t_ = *R*_v_ + *R*_d_, then becomes a minimum at diameter d* between the two limited states—the viscosity-limited state and the diffusion-limited state—as shown in Figure 5a.

Figure 5b shows the ascending velocity of a salt finger as a function of the diameter given by Equation (7). The velocity becomes a maximum at diameter d*, at which the total resistance, *R*_t_, becomes a minimum. We can solve the diameter, ascending velocity, and ascending flow rate at this maximum velocity state (d*v*/d*d* = 0) as follows:

(8) $$d^{*}=4\sqrt{\frac{\mu \;D_{s}}{\beta \;\delta_{v}\delta_{d}g}}\approx 1–5\;\mathrm{mm},$$

(9) $${v_{s}}^{*}=\;\frac{1}{2}\sqrt{\frac{\delta_{v}\;D_{s}\;g}{\mu \;\beta \;\delta_{d}}}\;C_{0}\approx 2–10\;\mathrm{mm}\;s^{-1},$$

(10) $$Q^{*}=2\pi \sqrt{\frac{\mu \;C^{2}{\,}_{0}\;D^{3}{\,}_{s}}{\beta^{3}\;\delta_{v}\delta^{3}{\,}_{d}\;g}}\approx 10–50\;{\mathrm{mm}}^{3}\;s^{-1},$$

where the asterisk denotes the value at the maximum velocity state and the numerical values are estimated from the parameter values used in the experiments: *C*_0_ ≈ 10–200 kg m^−3^, *μ* = 10^−3^ Pa s, *D*_s_ = 1.5 × 10^−9^ m^2^ s^−1^, *g* = 9.8 m s^−2^, *δ*_v_ = *δ*_d_ ≈ 0.03 mm, and *L* ≈ 0.1 m. The estimated values agree well with those observed for the salt fingers in our experiments (Figure 4). We also examined the ascending velocity and the diameter observed for a linear or meandering finger in each experiment and plotted them in Figure 5b on dimensionless scales (*v*_s_/*v*_s_* and *d*/d*). The mean value (open circle) and its standard deviation (error bars) show reasonable agreement with the state for the maximum velocity (solid circle), suggesting that the maximum velocity state is actually realized in the diameter and velocity for the linear and meandering fingers.

In Figure 3, we plot the ascending flow rate of a salt finger at the maximum velocity state, Q*, given by Equation (10), as a function of the initial salt density, *ρ*_init_ = *ρ*_w_ + *C*_0_, with *ρ*_w_ being the water density. We can see that when the injection flow rate is larger than this rate (*Q*_in_ > Q*), the finger diameter *d* becomes larger than d*, and the ascending motion is limited by salt diffusion. The finger then branches into two or more fingers via branching instability, thereby reducing the diffusive resistance and increasing the ascending velocity. Conversely, when *Q*_in_ < Q*, *d* becomes smaller than d*, and the ascending motion is limited by the viscosity. The finger then produces a beading pattern via beading instability, thereby reducing the viscous resistance and increasing the velocity. Both the instabilities and the associated morphological changes can be understood if salt fingers have a tendency to increase their ascending velocity under the prescribed non-equilibrium conditions.

### 3.2. Generation and Dissipation of Kinetic Energy

Let us discuss the reason for a salt finger’s tendency to increase the ascending velocity from a thermodynamic viewpoint. The kinetic energy of a salt finger is supplied by the work performed by the net buoyancy force (buoyancy minus gravity force), and this energy is dissipated into heat (internal energy) by the viscous drag force exerted on the moving finger. The rate of change of the kinetic energy, *E*_k_, per unit time per unit volume of a finger is given as follows:

(11) $$\frac{dE_{k}}{dt}=\;(\;f_{b}-f_{v})\;v\;=\;(C_{0}-C_{f})\;g\;v-R_{v}v^{2},$$

where *f*_b_ = (*C*_0_ − *C*_f_) *g* is the net buoyancy force, and *f*_v_ = *R*_v_ *v* is the viscous drag force exerted on the finger per unit volume. Using the salt balance Equation (3) and eliminating *C*_f_ in (11), we obtain the following equation:

(12) $$\frac{dE_{k}}{dt}=\;C_{0}\;g\;v-R_{t}v^{2}=G-D.$$

**Figure 6:**
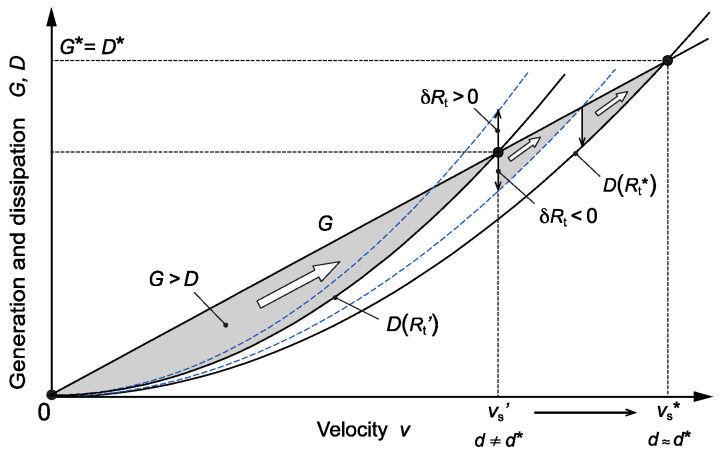
Generation rate, *G*, and dissipation rate, *D*, of kinetic energy as a function of the ascending velocity. In steady state, *G* = *D* and *v* = v_s_. A morphological change that reduces total resistance (*δR*_t_ < 0) tends to grow by a net supply of kinetic energy (*G* > *D*), whereas one that increases the resistance (*δR*_t_ > 0) cannot grow because of a deficit in kinetic energy (*G* < *D*), resulting in a final steady state with minimum resistance, *R*_t_*, maximum velocity, *v*_s_*, and maximum energy dissipation, D*.

The first term on the right-hand side, *G* = *C*_0_ *gv*, represents the total generation rate of kinetic energy and the second term, *D* = *R*_t_ *v*^2^, represents the total dissipation rate of kinetic energy due to viscosity and salt diffusion. The generation rate *G* increases with *v*, whereas the dissipation rate *D* increases with *v*^2^, as shown in Figure 6. At steady state (*G* = *D*), there exist two solutions: a static state with no motion (*v* = 0) and the steady ascending state (*v* = *v*_s_) described by Equation (7). The static state is unstable since any small positive fluctuation in velocity (*δv* > 0) leads to a net gain in kinetic energy (*G* > *D*), forming a positive feedback for the growth of the velocity. This growth can continue until a steady state (say, *v*_s_’ = *C*_0_ *g/R*_t_’), at which the energy balance is attained. This state is stable since any further fluctuations (± *δv*) lead to negative feedbacks: a positive fluctuation leads to *G* < *D*, whereas a negative fluctuation leads to *G* > *D*, thereby suppressing these fluctuations. A steady ascending motion thus tends to emerge in this system if the total resistance remains constant and the linear relation holds for the dissipative forces [22].

The total resistance, *R*_t_, however, varies depending on the size and shape of a finger. A morphological change that reduces the resistance (*δR*_t_ < 0) tends to grow by a net supply of kinetic energy (*G* > *D*). This growth can thus continue and lead to an increase in the velocity toward a new steady state (Figure 6). By contrast, a morphological change that increases the resistance (*δR*_t_ > 0) cannot grow but is suppressed since it reduces the energy supply (*G* < *D*). Because of this “ratchet-like” mechanism for energy accumulation, morphological changes under turbulent fluctuations always reduce the resistance and increase the velocity, resulting in a final steady state with minimum resistance (*R*_t_*) and maximum velocity (*v*_s_*), as demonstrated in our experiments. This final state also corresponds to the most active state at which the finger generates and dissipates kinetic energy at the maximal rate: G* = D* = *C*_0_ *gv*_s_*. The key process in the above mechanism is that a coherent or ordered structure, such as the beading or branching pattern, can only be formed by the net supply of work on the finger—work is the motive power for producing coherent structures [23].

### 3.3. Implications for Other Structure Formation Phenomena

It has long been suggested that the rate of energy dissipation, which is proportional to the rate of entropy production, tends to increase toward its maximum in various kinds of nonlinear, non-equilibrium systems. Examples include thermal convection [24], shear turbulence [24], atmospheric circulation [24,25,26,27,28], oceanic circulation [29], tropical cyclones [30], boundary layer turbulence [31], plasma convection [32], dendritic growth of crystals [6,7], vortex formation in granular flows [8], dendrite formation in voltage-driven beads [33], viscous finger formation in a Hele-Shaw cell [34,35], self-organization of microstructures [36], and biological evolution [37,38]. The energy dissipation rate in each system increases and reaches a maximum as the internal structure develops, while mechanical (available) energy is supplied from the non-equilibrium surroundings. This tendency has been referred to as the maximum entropy production (MEP) principle for nonlinear, non-equilibrium systems [39,40,41,42,43,44,45], which shows a sharp contrast to the principle of minimum entropy production suggested for linear processes in near-equilibrium systems [2,10,11,46]. While the basic theoretical background of this MEP principle is still debatable, the key factor seems to be related to the dynamic or morphological instability inherent in far-from-equilibrium systems (e.g., Sec. 7.2 in [40]). For example, Martyushev and Birzina [34,35] investigated morphological instability in the initial growth of an interface between two pressurised fluids in a gap between two parallel plates (a Hele-Shaw cell). They found that the critical condition for the initial growth of viscous fingers estimated by the state of larger entropy production was in reasonable agreement with the experimental results [35]. Although stability analysis provides useful insight into the initial growth condition of viscous fingers, it cannot state the situation of the final steady state in which actual emerging fingers converge after their growth. We have shown in this paper that a salt finger produces a finger pattern that reduces the flow resistance and increases the ascending velocity by enhancing the energy supply from the non-equilibrium surroundings. The energy dissipation rate reaches a maximum at the final steady state, and the dissipated energy, as well as the produced entropy, is discharged steadily into its surroundings, thereby bringing the surroundings to equilibrium at the fastest rate [24,40]. The “ratchet-like” mechanism for energy accumulation found for the nonlinear growth of a salt finger seems to work also for other structure formation phenomena in highly non-equilibrium systems [1,2,3,4,5,6,7,8,24,25,26,27,28,29,30,31,32,33,34,35,36,37,38,39,40,41,42,43,44,45]. Further studies are needed to verify this mechanism for other phenomena, and some attempts will be reported in future publications.

The proposed mechanism for beading and branching pattern formation seems to apply also to the growth and division of protocells in the primordial oceans of the Earth. Oparin [47] suggested that organic-rich liquid emulsion droplets, called “coacervates,” formed via phase separation from a mixture of organic materials and seawater in the primordial oceans. Numerical simulations of such coacervate droplets showed that they can form, elongate, and divide when super-saturation of the chemical component that fuels them exceeds critical values [48]. Elongation occurs at the periphery of a growing droplet where the concentration of the chemical fuel is high. The diameter of curvature at the periphery becomes larger than the optimum growth size (*d* > d*_peri_) and thus leads to protrusion of the surface by Mullins–Sekerka instability [49]. By contrast, division takes place at the central waistline of an elongated droplet where the concentration is low and the diameter is smaller than the optimum size (*d* < d*_cent_), leading to surface shrinkage and division. The two morphological transitions are therefore essentially identical to the branching and beading instabilities (Figure 5b) under the inhomogeneous concentration field produced around a growing droplet. The diffusion flux of the chemical fuel is higher in the outer regions than in the vicinity of the droplets. Each droplet thus migrates toward the chemically rich outer regions while repeating elongation and division [48]. If organic-rich chemical fuels were continuously supplied to seawater from the surroundings by photochemical reactions due to solar radiation or hydrothermal fluids supplied from volcanic activities, coacervate droplets could have formed via phase separation, and they would have grown, elongated, and divided by adjusting their shapes so as to increase the growth rate and free energy dissipation. Growth competition among these droplets could have eventually led to the emergence of primary protocells that dissipated free energy and produced entropy most efficiently [37,50], whereby the state of the surrounding system was brought to equilibrium at the maximum rate.

It should be noted that actual protocells might have been composed of complex materials such as amphipathic lipids and nucleic acids [51,52]. However, even simple emulsion droplets (coacerbates) could grow, elongate, and divide under the effect of surface tension and the diffusion of chemical fuels under non-equilibrium conditions [48,53]. The beading and branching instabilities of a salt finger can thus be seen as a simple, dynamic compartment model for the growth and development of primordial protocells. Further experimental studies could provide valuable insights into what is currently unknown about the early evolution of protocells.

## 4. Concluding Remarks

We have shown in this paper that two new types of morphological transitions, beading and branching instabilities, are caused by a local accumulation of available energy, which minimizes the flow resistance and maximizes the energy dissipation in the final steady state. The emergence and evolution of ordered structures therefore accelerate the rate at which the surrounding non-equilibrium system approaches equilibrium. The same energy accumulation mechanism-induced structure formation is likely to occur in non-equilibrium systems on Earth and other planets that exchange radiant energy with the surrounding universe composed of the hot sun and cold space. We suggest that all of these structures, as well as living organisms, are formed so as to produce entropy and equilibrate the highly non-equilibrium universe.

## Data Availability

All data are available in the main text or the Supplementary Materials.

## Supplementary Materials

The following supporting information can be downloaded at: https://www.mdpi.com/article/10.3390/e27020106/s1, Videos S1–S5: movies of five typical salt finger patterns (S1: beading, S2: linear, S3: meandering, S4: branching, S5: multiple branching).

## Appendix A. Experimental Methods

A schematic representation of the experimental apparatus is shown in Figure 2h. A transparent container, 0.1 m × 0.1 m × 0.2 m high, contained solution B (sucrose solution). Solution A (salt solution) of the same density was supplied from a syringe (30 × 10^3^ mm^3^) at a constant flow rate, *Q*_in_ = 0.17–100 mm^3^ s^−1^, using a syringe driver. Using a floating hydrometer, we adjusted the initial densities of the solutions to the same value, *ρ*_init_ = 1010, 1050, 1100, or 1200 kg m^−3^, with an accuracy of ± 0.5 kg m^3^. The salt solution was dyed blue to make the fingers clearly visible. A porous stone was attached to the injection point to reduce the initial flow velocity and avoid initial disturbances. After trying several materials of different sizes and shapes, we chose a porous stone (diameter: 10 mm; length: 18 mm), as it most efficiently reduced the initial flow disturbances and the associated turbulence.

At the beginning of each experiment, the porous stone was filled and saturated with the salt solution using the syringe driver. At this stage, no sucrose solution was added to the container. When the salt solution flowed out from the surface of the porous stone, which was visible as a thin layer of dyed fluid covering the surface, the injection was stopped, and the sucrose solution was added slowly to the container so as not to mix it with the salt solution filled in the stone. After this procedure, the injection was started again at a constant rate (*Q*_in_) using the syringe driver. An accumulation of the salt solution was observed around the porous stone, and shortly thereafter, an ascending motion of a finger-like plume was caused by buoyancy force due to preferential diffusion of salt into the surrounding sucrose solution.

The first part of the ascending plume usually consisted of a group of small fingers in a rather complicated shape resembling a “hydra.” This assemblage, however, was soon followed and overtaken by a faster and steady “stem”. Aproximately 30 s after the start of injection, the finger shape and its motion reached a nearly steady state. We observed the shape, diameter, and ascending velocity of such a salt finger in its steady state with two digital video cameras; one was placed in front of the container and the other was placed to the side. The digital images were transferred to a computer, and the diameter and velocity were measured at 5 cm above the porous stone by tracing the finger motion observed with the front camera. In the case of multiple branching fingers, a typical finger rising on the side was selected for measurement to avoid overlapping of finger images. In this case, images from the side camera were used to ensure that the selected finger position was within ± 1 cm in front or behind the position of the porous stone, thereby reducing measurement errors due to parallax. The measurement was repeated at least 10 times (10–20 times) to obtain the mean value and its standard deviation. We changed the injection flow rate, *Q*_in_, after the steady-state measurements and repeated the procedure for the new steady state. The results of the diameter and velocity measurements on a total of 26 steady-state fingers for four different initial densities are shown in Figure 4 and Table A1.

It should be noted that the viscosity of a salt solution is generally lower than that of a sucrose solution of equal density (see the footnote of Table A1). In this case, we may expect the Saffman–Taylor instability [54] that is known to occur when two pressurized fluids with different viscosities coexist in a porous medium, such as a Hele–Shaw cell [34,35]. In our experiments, however, the porous stone was saturated with salt solution at the beginning of each experiment. The sucrose solution outside the porous stone was unconstrained and in contact with the free atmosphere. Thus, Saffman–Taylor instability was unlikely to occur. We confirmed this through experiments, in which polyethylene glycol was added to a salt solution to make its viscosity the same as that of a sucrose solution of equal density (1100 kg m^−3^). Exactly the same results as those found in our experiments (Figure 2 and Figure 3) were observed in the same viscosity experiments, eliminating the possibility of Saffman–Taylor instability in our case. However, Saffman–Taylor instability should play an important role in the pattern formation of fluids under constrained conditions such as a Hele–Shaw cell [34,35,54].

The ascending flow rate of a single finger was calculated from the measured diameter, *d*, and velocity, *v*, as *Q* = *π* (*d*/2)^2^ *v*. The standard error of the flow rate, Δ*Q*, could then be estimated by the following equation:

(A1) $$\Delta Q\;=\;\left|\frac{\partial Q}{\partial d}\right|\Delta d+\left|\frac{\partial Q}{\partial \upsilon }\right|\Delta v\;=\;\frac{\pi }{2}dv\;\Delta d+\frac{\pi }{4}d^{2}\;\Delta v,$$

where Δ*d* and Δ*v* are the standard deviations of *d* and *v*, respectively. The estimated standard errors of the flow rates from Equation (A1) are shown as error bars in Figure 4c.

**Table A1:** Results of the salt finger experiments. *ρ*_init_, initial density; *Q*_in_, injection flow rate; *v*, velocity; *d*, diameter of a salt finger. Finger types: Bd, beading; L, linear; M, meandering; B, branching; MB, multiple branching types. If a finger exhibits a bimodal mixture of two types, both types are described. The mean velocity and diameter of linear or meandering fingers designated by the cross (+) are shown in Figure 5b on dimensionless scales.

*ρ*_init_ ^1^ (kg m^−3^)	*Q*_in_ (mm^3^ s^−1^)	*v* (mm s^−1^)	*d* (mm)	Finger Type
1010	0.83	1.91 ± 0.37	2.02 ± 0.30	Bd
1010	1.67	1.53 ± 0.13	2.74 ± 0.30	Bd, M (+)
1010	8.33	2.19 ± 0.48	2.31 ± 0.53	B, MB
1010	16.7	1.67 ± 0.41	1.51 ± 0.27	MB
1050	0.83	3.85 ± 0.32	2.92 ± 0.37	Bd
1050	1.67	4.90 ± 0.57	3.03 ± 0.22	Bd
1050	3.33	7.54 ± 0.81	3.04 ± 0.38	Bd (+)
1050	5.0	6.82 ± 0.84	2.95 ± 0.37	B
1050	8.33	6.29 ± 0.65	3.46 ± 0.47	MB
1050	66.7	6.77 ± 0.94	2.20 ± 0.45	MB
1050	100	5.76 ± 0.62	2.49 ± 0.43	MB
1100	0.83	5.25 ± 0.76	1.90 ± 0.22	Bd
1100	1.67	5.51 ± 0.66	1.57 ± 0.18	L (+)
1100	5.0	8.40 ± 0.65	1.85 ± 0.35	M
1100	8.33	9.25 ± 0.52	2.17 ± 0.28	M
1100	12.5	11.97 ± 1.59	1.79 ± 0.27	M
1100	16.7	8.95 ± 0.67	1.18 ± 0.28	B
1100	33.3	7.84 ± 1.50	1.55 ± 0.22	B
1100	50.0	8.34 ± 1.64	1.08 ± 0.14	MB
1100	66.7	9.40 ± 1.40	1.25 ± 0.26	MB
1100	83.3	7.25 ± 1.42	1.48 ± 0.32	MB
1200	1.67	3.06 ± 0.28	1.81 ± 0.29	M
1200	8.33	4.71 ± 0.62	2.64 ± 0.29	M (+)
1200	16.7	5.80 ± 1.12	1.58 ± 0.36	B, MB
1200	50.0	6.81 ± 1.04	1.55 ± 0.26	MB
1200	83.3	7.06 ± 1.74	1.72 ± 0.33	MB

^1^ The viscosities of the salt and sucrose solutions are estimated to be 1.00, 1.10, 1.31, 1.96 and 1.01, 1.42, 2.49, 8.76 mPa s, respectively, for densities *ρ*_init_ = 1010, 1050, 1100, 1200 kg m^–3^ at a temperature of 293 K [55,56].
